# Tumor Mesenchymal Stem-Like Cell as a Prognostic Marker in Primary Glioblastoma

**DOI:** 10.1155/2016/6756983

**Published:** 2016-02-11

**Authors:** Seon-Jin Yoon, Jin-Kyoung Shim, Jong Hee Chang, Ju Hyung Moon, Tae-Hoon Roh, Kyoung Su Sung, Ji-Hyun Lee, Eui-Hyun Kim, Sun Ho Kim, Yong-Kil Hong, Su-Jae Lee, Yong-Min Huh, Seok-Gu Kang

**Affiliations:** ^1^Department of Neurosurgery, Brain Tumor Center, Severance Hospital, Yonsei University College of Medicine, 50-1 Yonsei-ro, Seodaemun-gu, Seoul 120-752, Republic of Korea; ^2^Department of Neurosurgery, Seoul St. Mary's Hospital, The Catholic University of Korea College of Medicine, 222 Banpo-daero, Seocho-gu, Seoul 137-701, Republic of Korea; ^3^Department of Life Science, Hanyang University, 17 Haendang-dong, Seongdong-gu, Seoul 133-791, Republic of Korea; ^4^Department of Radiology, Severance Hospital, Yonsei University College of Medicine, 50-1 Yonsei-ro, Seodaemun-gu, Seoul 120-752, Republic of Korea

## Abstract

The isolation from brain tumors of tumor mesenchymal stem-like cells (tMSLCs) suggests that these cells play a role in creating a microenvironment for tumor initiation and progression. The clinical characteristics of patients with primary glioblastoma (pGBM) positive for tMSLCs have not been determined. This study analyzed samples from 82 patients with pGBM who had undergone tumor removal, pathological diagnosis, and isolation of tMSLC from April 2009 to October 2014. Survival, extent of resection, molecular markers, and tMSLC culture results were statistically evaluated. Median overall survival was 18.6 months, 15.0 months in tMSLC-positive patients and 29.5 months in tMSLC-negative patients (*P* = 0.014). Multivariate cox regression model showed isolation of tMSLC (OR = 2.5, 95% CI = 1.1~5.6, *P* = 0.021) showed poor outcome while larger extent of resection (OR = 0.5, 95% CI = 0.2~0.8, *P* = 0.011) has association with better outcome. The presence of tMSLCs isolated from the specimen of pGBM is associated with the survival of patient.

## 1. Introduction

Glioblastomas (GBMs) are generated by interactions between cancer stem cells (CSCs) and stroma [1–3]. The accumulation of molecular errors in CSCs initiates tumorigenesis, and these CSCs aggregate with stromal cells, which synergistically aggravate the disease [4, 5]. Mesenchymal stem-like cells (MSLCs) have been isolated from normal brain [6, 7] and Lang et al. [8] presented the isolation of mesenchymal stem cells (MSCs) from glioma for the first time. In addition, tumor MSLCs (tMSLCs) have been isolated from several human brain tumors [2, 9–11], suggesting that these cells play a role in creating a microenvironment conducive to brain tumor initiation and progression [2, 12–14].

GBMs can be grouped into several subtypes, based on molecular markers, gene expression profiles [15–19], and chromosomal aberration [20, 21]. Based on their genetic characteristics, GBMs can be divided into four types, with the mesenchymal type having the poorest prognosis [17, 18, 22]. Several molecular markers have been shown to be related to survival benefits in patients with GBM, including O-6-methylguanine-DNA methyltransferase (MGMT) methylation and the isocitrate dehydrogenase (IDH) 1/2 mutation [23–25]. The prognostic value of heterozygosity (LOH) at chromosomes 1p and 19q, however, is unclear [26, 27]. Isolation of CSCs from primary GBM (pGBM) samples can also predict the natural course of pGBM [28]. Although tumor stromal cells were found to have a significant impact on patient survival [29, 30], the clinical significance of isolation of tMSLCs, a type of stromal cells, from pGBM stroma has not been determined.

We hypothesized that the presence of tMSLCs may aggravate the natural course of pGBM. This study therefore assessed whether the presence of tMSLCs in pGBM patients has an effect on patient survival and prognosis.

## 2. Materials and Methods

### 2.1. Patient Information

A total of 82 patients with pGBM who received standard therapy [31] at two institutions (Severance Hospital, Yonsei University College of Medicine, and Seoul St. Mary's Hospital, the Catholic University of Korea College of Medicine) from April 2009 to October 2014 were included in this study (Table 1). We followed up the cohort from previous report [11] and added new additional patients that were not included in that report [11]. Approval for harvest and investigation was obtained from the institutional review boards of the two institutions, and all patients provided written informed consent, as specified in the Declaration of Helsinki. Specimens for isolation of tMSLCs were collected in the operating theater from patients undergoing surgery. All surgical specimens were evaluated by two neuropathologists, who diagnosed each patient according to World Health Organization (WHO) classifications [32]. Survival, extent of resection, molecular markers, and tMSLC culture results were analyzed statistically. Inclusion criteria were as follows: the first pathologic diagnosis of primary glioblastoma patients with the standard Stupp protocol; radiation dose of 60 Gy fractionated by 30 times; and Stupp protocol within 2 weeks after the pathological diagnosis. Excluded patients met following criteria: recurring glioblastoma after previous surgery; gliosarcoma; nonstandard dose for temozolomide administration; hypofractionated radiotherapy; and poor hematologic profile that delayed normal course of standard treatment.

### 2.2. Initial Treatment

All patients underwent surgical resection, aimed at gross total resection of the tumor, followed by concurrent chemotherapy and radiotherapy and adjuvant chemotherapy [31]. Gross total tumor resection was defined as macroscopic removal of 100% or above of the tumor mass found on magnetic resonance (MR) T1 enhanced and T2 images [33, 34]. Patients not suitable for total resection underwent subtotal resection, defined as removal of macroscopic tumor volume ≥90% but <100%, or partial resection, defined as removal of macroscopic tumor volume <90%. The extent of tumor resection was estimated and classified by the neurosurgeons and rechecked by postoperative review of MR imaging (MRI) scans. All patients received postoperative adjuvant radiotherapy with concomitant and adjuvant temozolomide (TMZ), as described previously [31]. Each patient was offered standard therapy [31] after pathologic confirmation. The only factors determining the continuation of standard treatment were patient tolerance (general condition, laboratory abnormalities such as hematologic problems), family agreement for the treatment, and patient survival. The correlation between molecular markers (MGMT methylation, p53, 1p LOH, 19q LOH, Ki67 index, and IDH1 mutation) and survival was analyzed statistically. MGMT methylation was assessed by polymerase chain reaction (PCR) and LOH at chromosomes 1p and 19q by fluorescent in situ hybridization (FISH).

### 2.3. Isolation of tMSLCs

tMSLCs with characteristics similar to MSCs have been isolated from brain tumor specimens [9–11]. Specimens from patients with GBM were obtained from the operating room. Briefly, cells were isolated during tumor removal using a mechanical dissociation method within 1 h. Surgical specimens were minced and dissociated with a scalpel in minimal essential medium-*α* (MEM*α*; Mediatech, Herndon, VA, USA) and passed through a series of cell strainers with a 100 *μ*m nylon mesh. Cell suspensions were washed twice in MEM*α* and then cultured in complete MSC medium consisting of MEM*α*, 10% fetal bovine serum (FBS; Gibco, Invitrogen), 2 mM L-glutamine (Mediatech), and 1x antibiotic-antimycotic solution (Invitrogen, Carlsbad, CA, USA). After 24 h, nonadherent cells were removed by washing twice with phosphate-buffered saline (PBS; Mediatech), and the adherent cells were cultured until they reached confluence. The cells were then trypsinized (0.25% trypsin with 0.1% EDTA) and subcultured at a density of 5 × 10^3^ cells/cm^2^. Isolated cells were evaluated for several mesenchymal features, including plastic adhesion, trilineage differentiation, the presence of typical MSC surface markers (positive for CD 105, CD 90, and CD 72 and negative for CD 45, CD 31, and NG2), and nontumorigenic behavior [11, 13, 35].

### 2.4. Statistical Analysis

The primary outcome measure was median overall survival (OS), defined as the interval from date of surgery confirming the diagnosis of pGBM to the date of last follow-up visit or death [36]. Immunohistochemical analysis of p53 expression was defined as immunopositivity when the areas with staining of ≥50% of cancer cells were found. Ki 67 index was defined as immunopositive when the stained area exceeded 10% or more. Among clinically deemed primary glioblastomas, 5 patients (6%) had mutation on IDH1. They were not excluded from our study for comprehensive evaluation. Because of small number of patients with IDH1 mutation and previous reports about different clinical characteristics [37], they were not eligible for survival analysis and excluded from multivariate cox regression model. Survival was analyzed by the Kaplan-Meier method and compared by the log-rank test. Demographic characteristics were compared using Fisher's exact test or *t*-test. All statistical analyses were performed using SPSS 22 (IBM Korea, Seoul, Korea), with *P* values less than 0.05 regarded as statistically significant.

## 3. Results

### 3.1. Patient Characteristics

Of the 82 patients with pGBM, 48 (59%) were positive and 34 (41%) negative for tMSLCs (Table 1), with no group differences in the extent of resection (*P* = 0.471), age (*P* = 0.683), and expression of specific molecular markers (IDH1, *P* = 0.642; MGMT promoter, *P* = 0.653; p53, *P* = 0.522 for positivity *P* = 0.492 for the percentage of immunohistochemistry; EGFR, *P* = 0.161; Ki 67, *P* = 0.739 for the number of each of the immunostaining statuses *P* = 0.057 for the percentage of immunohistochemistry). All of 48 tMSLCs isolated from specimens showed trilineage differentiation, expression of MSC surface markers, and adherence to a plastic plate without gliomagenesis.

### 3.2. Patient Survival

**Figure 1:**
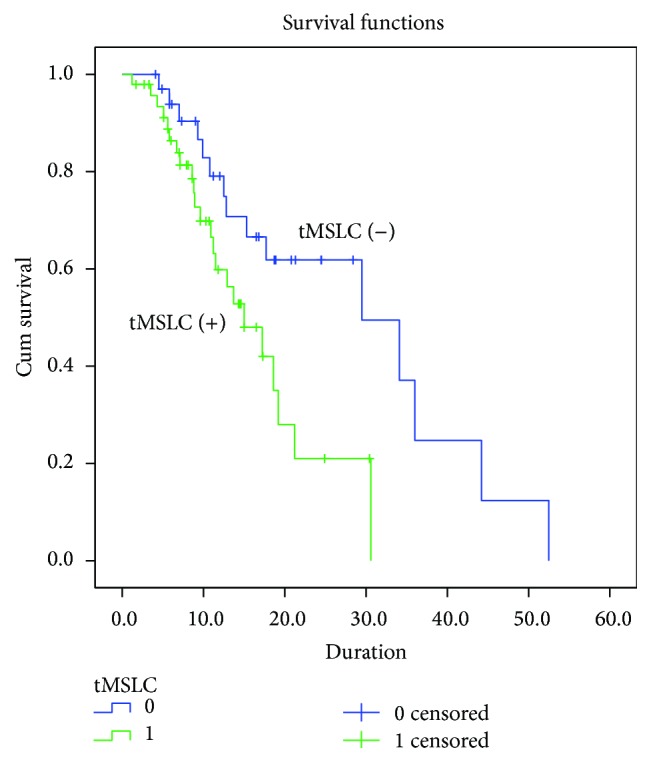
Kaplan-Meier estimates of overall survival according to tMSLCs isolation (*P* = 0.014 as calculated by the log-rank test).

At a medium follow-up of 11.1 months, 38 patients died. The median survival duration of all pGBM patients was 18.6 months, 15.0 months in patients positive for tMSLCs and 29.5 months in patients negative for tMSLCs (*P* = 0.014; Figure 1). The 6-, 12-, and 24-month actuarial rates were 86%, 60%, and 21%, respectively, in patients positive for tMSLCs and 93%, 79%, and 61%, respectively, in patients negative for tMSLCs. From univariate cox proportional regression, the only factor associated with poor survival was isolation of tMSLCs from the specimen (OR = 2.4, 95% CI = 1.2~5.1, *P* = 0.017; Table 2). We included extent of resection, codeletion of 1p19q, MGMT methylation, and Ki 67 index to a multivariate cox model with a result that isolation of tMSLCs (OR = 2.5, 95% CI = 1.1~5.6, *P* = 0.021) was associated with poorer outcome and larger extent of resection had association with better prognosis (OR = 0.5, 95% CI = 0.2~0.8, *P* = 0.011) while codeletion of 1p19q, MGMT methylation, and Ki 67 index does not differentiate survival of patients.

## 4. Discussion

MSLCs are cells with MSC-like properties that have been isolated from brain [6, 7], especially from brain tumors (tMSLCs) [2, 9–11, 13, 38]. These cells possess MSC surface antigens, show trilineage differentiation, adhere to plastic plates, and are nontumorigenic [11, 13, 35].

This study found that OS differed significantly between pGBM patients positive and negative for tMSLCs, suggesting that tMSLCs may play a role in the progression of pGBM. Although the “seed and soil” concept of cancer biology was proposed more than 125 years ago [39–41], GBM tumorspheres (TS) have been demonstrated recently [42, 43], with tMSLCs being a factor in this concept [9–12]. Although the exact function of tMSLCs in pGBM is not well understood, this study showed that tMSLCs were clinically important, in that they were prognostic of survival. The next step should be to assess the interactions between the GBM TS and tMSLCs.

Our overall survival (18.6 months) was higher than previously reported paper [31]. Although we described gross total resection in this paper, we indeed did supratotal resection, which made longer overall survival in our patients; however it was not proved through publication. The small number of patients (82 cases in total) recruited to this retrospective study is a limitation to interpret the results of this study. Although we compiled as many patients as possible, only subgroup that meets strict inclusion and exclusion criteria has limited number of patients. As our analysis does not show different prognosis in overall survival that was determined by MGMT methylation or LOH of 1p19q which was shown in more inclusive larger patient set, cautious interpretation of our result is required. In addition, our study included IDH1 mutant patients and unknown patients, although we described pGBM. IDH1 mutant patients should be secondary GBM but these patients were allocated to both groups without statistical significance, so we included all IDH1 mutants patients and unknown patients in this study.

From original group of 82 patients, only five of 70 patients tested (7.1%) had IDH1 mutations, including one from 27 tMSLC-negative (3.7%) patients and four from 43 tMSLC-positive (9.3%) patients. While these 5 tumors were not clinically suspected as secondary glioblastomas, they were excluded from multivariate cox regression model as the entity was shown to have different prognosis [37]. Patients with mutated IDH1 were younger than those with wild type IDH1 (45.3 versus 60.7 years) [37]. Although LOH at 1p or 19q was found to correlate with longer OS in patients with oligodendroglioma [44], the association in patients with pGBM remains unclear. In our result, codeletion of 1p19q was not associated with prognosis (univariate: OR = 1.3, 95% CI = 0.6~3.0, *P* = 0.532; multivariate: OR = 1.1, 95% CI = 0.4~2.6, *P* = 0.869). Analysis of MGMT promotor showed that 42% of specimens were methylated (41% in tMSLCs (+), 47% in tMSLCs (−)) and that median OS tended to be longer in patients with methylation while lacking statistical significance (18.6 versus 15.0 months, *P* = 0.650). IHC analysis of p53 found that 28% of specimens were stained for this marker. However, median OS was similar in p53 positive and negative patients (18.6 versus 13.7 months, *P* = 0.415).

In this study, tMSLCs were isolated from 58.5% of patients with pGBM, compared with 46.2% in a previous study [11]. This increase, despite using the same isolation method, reemphasizes that isolation of a specific cell type from a tumor specimen requires a standardized method or may reflect a learning curve among laboratory staffs. Further research may identify specific cell markers prognostic of OS. We present novel findings of tMSLCs as a prognostic marker. Several noteworthy publications have discussed potential roles for MSLCs in glioma natural history, such as increase in angiogenesis [12] or increased proliferation and self-renewal of glioma stem cells [13]. Although we could introduce possible mechanisms based on published articles [12, 13], this study could not show any direct biological mechanism of tMSLCs as prognostic markers, which we believe one of the limitations of current study.

Mesenchymal features may contribute to poor survival in patients with brain tumors. Higher grade gliomas [11] and meningiomas [10] are more likely to be identified to have tMSLCs. Indeed, tMSLCs could not be isolated from WHO grade 1 gliomas and meningiomas, whereas 20%, 33%, and 32% (or 46.2% without secondary GBM and recurrent GBM) of WHO grade 2, 3, and 4 gliomas, respectively, were positive for tMSLCs [11]. It remains unclear, however, how the presence of tMSLCs aggravates the natural history of a brain tumor or contributes to tumor progression.

## 5. Conclusion

Isolation of tMSLCs is associated with the survival of pGBM patients. tMSLCs may have a critical role in the survival of patients with pGBM. Other cell types may predict the clinical course of patients with pGBM. In addition, cell surface markers and molecular markers of pGBM may have prognostic value, and the interactions of tMSLCs with gCSCs may better reveal the role of these cell types in pGBM patients.

## Figures and Tables

**Table 1:** Demographic and clinical characteristics of patients with pGBM.

Characteristics	tMSLCs (+) (*N* = 48)	tMSLCs (−) (*N* = 34)	*P* value
Age (years)			0.683
Median	57.5	61.0	
Range	28~85	24~80	
Age, no. (%)			0.899
<50 years, no. (%)	9 (19)	6 (18)	
≥50 years, no. (%)	39 (81)	28 (82)	
Gender			0.110
Male, no. (%)	33 (69)	17 (50)	
Female, no. (%)	15 (31)	17 (50)	
Median survival (months)	15.0	29.5	0.014
95% CI	9.6~20.4	11.9~47.1	
Pathological diagnosis	pGBM	pGBM	
Treatment	OP/Stupp	OP/Stupp	
Extent of operation (patients)			0.471
Gross total resection (≥100%)	29 (60)	19 (56)	
Subtotal resection (90% ≤, <100%)	18 (38)	12 (35)	
Partial resection (<90%)	1 (2)	3 (9)	
Molecular markers			
IDH1			0.642
Wild type, no. (%)	39 (91)	26 (96)	
Mutation, no. (%)	4 (9)	1 (4)	
Missing data, no. (%)	5 (10)	7 (21)	
1p19q			0.341
No codeletion, no. (%)	37 (80)	30 (91)	
Median survival (months)	15.0	29.5	0.011
95% CI	8.9~21.1	9.1~50.0	
Codeletion, no. (%)	9 (20)	3 (9)	
Median survival (months)	12.9	9.3	0.886
95% CI	0.8~25.0	1.6~17.0	
Missing data, no. (%)	2 (4)	1 (3)	
MGMT promoter			0.653
Wild type, no. (%)	27 (59)	18 (53)	
Median survival (months)	15.0	NA	0.122
95% CI	8.8~21.2	NA	
Methylated, no. (%)	19 (41)	16 (47)	
Median survival (months)	18.6	34.1	0.164
95% CI	6.2~31.0	13.6~54.6	
Missing data, no. (%)	2 (4)	0 (0)	
p53			0.522
IHC negative (<50%), no. (%)	23 (77)	13 (65)	
Median survival (months)	13.7	NA	0.324
95% CI	9.8~17.6	NA	
IHC positive (≥50%), no. (%)	7 (23)	7 (35)	
Median survival (months)	18.6	NA	0.704
95% CI	9.9~27.3	NA	
IHC mean ± S.D.	28.4 ± 27.0	33.9 ± 28.8	0.492
Range (%)	1.5~85	2.5~90	
Missing data, no. (%)	18 (38)	14 (41)	
EGFR			
IHC mean ± S.D.	1.9 ± 1.1	2.3 ± 0.9	0.161
Range	0~3	0~3	
Missing data, no. (%)	6 (13)	7 (21)	
Ki 67			0.739
IHC negative (<10%), no. (%)	5 (11)	3 (9)	
IHC positive (≥10%), no. (%)	40 (89)	31 (91)	
IHC % mean ± S.D.	22.6 ± 14.1	30.1 ± 20.2	0.057
Range (%)	2~60	3~80	
Missing data, no. (%)	3 (6)	0 (0)	

S.D.: standard deviation; IDH: isocitrate dehydrogenase; LOH: loss of heterozygosity; OP/Stupp: operation followed by Stupp's regimen; MGMT: O-6-methylguanine-DNA methyltransferase; EGFR: epidermal growth factor receptor; NA: not available.

**Table 2:** Cox proportional hazard regression model of factors prognostic of overall survival in patients with pGBM.

Characteristics	Univariate			Multivariate		
	OR	95% CI	*P* value	OR	95% CI	*P* value
Isolation of tMSLCs	2.4	1.2~5.1	0.017	2.5	1.1~5.6	0.021
Age ≥ 50 years	1.3	0.5~3.1	0.587			
Extent of resection	0.7	0.4~1.1	0.151	0.5	0.2~0.8	0.011
IDH1 mutation	1.6	0.4~6.7	0.551			
LOH 1p19q	1.3	0.6~3.0	0.532	1.1	0.4~2.6	0.869
MGMT methylation	0.8	0.4~1.6	0.522	0.9	0.4~1.9	0.792
p53 ≥ 50%	0.7	0.2~1.8	0.418			
EGFR	1.1	0.7~1.5	0.782			
Ki 67 index ≥ 10%	3.3	0.5~24.7	0.235	5.4	0.7~42.0	0.107

tMSLCs: tumor mesenchymal stem-like cells; IDH: isocitrate dehydrogenase; LOH: loss of heterozygosity; MGMT: O-6-methylguanine-DNA methyltransferase; EGFR: epidermal growth factor receptor; OR: odds ratio; CI: confidence interval.
